# Microbiome modulation and behavioural improvements in children with fragile X syndrome following probiotic intake: A pilot study

**DOI:** 10.1038/s41598-025-29896-1

**Published:** 2025-12-05

**Authors:** Dragana Protic, Danijela Bascarevic, Sanja Dimitrijevic, Jovan Pesovic, Vladimir Nikolic, Sasa Nikolic, Velibor Novicevic, Jovana Markovic, Irena Arandjelovic, Dusanka Savic-Pavicevic, Margo Diricks, Meriem Belheouane, Matthias Merker

**Affiliations:** 1 Department of Pharmacology, Clinical Pharmacology and Toxicology, Faculty of Medicine, University of Belgrade, Belgrade, Serbia; 2 Special Hospital for Cerebral Palsy and Developmental Neurology, Belgrade, Serbia; 3 Institute for Rehabilitation, Belgrade, Serbia; 4 Faculty of Biology, Center for Human Molecular Genetics, University of Belgrade, Belgrade, Serbia; 5 Institute of Epidemiology, Faculty of Medicine, University of Belgrade, Belgrade, Serbia; 6 Institute of Microbiology and Immunology, Faculty of Medicine, University of Belgrade, Belgrade, Serbia; 7 Molecular and Experimental Mycobacteriology Research Center Borstel, Borstel, Germany; 8 Leibniz Research Alliance INFECTIONS, Borstel, Germany; 9 Evolution of the Resistome, Research Center Borstel, Borstel, Germany; 10 German Center for Infection Research (DZIF), Partner Site Hamburg- Lübeck-Borstel-Riems, Lübeck, Borstel, Germany

**Keywords:** *FMR1* gene, Fragile X syndrome, Probiotics, Behaviour, Microbiome, Gut-brain axis, Microbiology, Neuroscience

## Abstract

The gut microbiome (GM) is increasingly recognized as a key modulator of neurodevelopment via the microbiome-gut-brain axis. Fragile X syndrome (FXS), the most common inherited monogenic cause of intellectual disability, shares behavioural and molecular features with other neurodevelopmental disorders (NDDs), yet the role of the GM in FXS remains largely unexplored. In this open-label, single-arm trial, 15 children with genetically confirmed FXS received a daily probiotic formulation containing *Lactobacillus casei*, *Lactobacillus salivarius*, and *Bifidobacterium breve* for 12 weeks. Behavioural analysis and metagenomic sequencing with network and pathway analyses were performed before and after probiotic supplementation. Significant improvements were observed in irritability (-3.9, SD: ± 5.2; *p* = 0.027), communication (+ 1.7, SD: ± 2.5; *p* = 0.022), socialization (+ 1.4, SD: ± 2.1; *p* = 0.033), and adaptive behaviour (+ 1.3, SD: ± 1.4; *p* = 0.004). While overall microbial diversity remained stable, SparCC network analysis revealed increases in connectivity measures such as edge count and clustering coefficient, indicating denser microbial interactions and greater community coordination after probiotic supplementation. Functional profiling showed trends toward increased microbial activity in fatty acid biosynthesis, NAD salvage, and starch degradation pathways. This pilot study provides initial evidence that probiotics may modulate structural and functional properties of the GM, with potential links to improved behavioural outcomes in children with FXS. Larger, controlled trials are needed to validate the therapeutic potential of GM-targeted interventions in FXS and related NDDs.

**Supplementary Information:**

The online version contains supplementary material available at 10.1038/s41598-025-29896-1.

## Introduction

The microbiome-gut-brain axis plays a crucial role in neurodevelopment and brain function. Emerging evidence highlights the importance of the gut microbiota (GM) to human health, which begins to form in early childhood and is shaped throughout life by factors such as diet, medications, and stress^1^. The GM affects human behaviour through multiple pathways, including the nervous system, immune system, tryptophan metabolism, and the hypothalamic-pituitary-adrenal (HPA) axis^2^. These effects are mediated by microbial metabolites, neurotransmitter production, and metabolic processes and can be linked to neurodevelopmental disorders^3^. The GM synthesizes key neurotransmitters like gamma-aminobutyric acid (GABA), dopamine, histamine, and serotonin. While some of those cannot cross the blood-brain barrier, the GM provides precursor molecules (e.g., phenylalanine, tyrosine, tryptophan) essential for neurotransmitters’ synthesis^4^. This highlights its role in cognitive function, behaviour, and neuronal plasticity through neurotransmitter pathways^4–6^. However, the precise role of the GM in modulating neurodevelopmental disorders (NDDs) symptoms remains a subject of ongoing debate.

Neurodevelopmental disorders are diverse and complex, and they share overlapping molecular and behavioural features^7^. Among them, fragile X syndrome (FXS) stands out as the most common monogenic cause of both intellectual disability (ID) and autism spectrum disorder (ASD)^8,9^. FXS results from CCG expansions of > 200 repeats (full mutation) in the *FMR1* gene, leading to methylation-induced gene silencing and deficiency of the fragile X messenger ribonucleoprotein (FMRP) which is required for normal brain development and function^10,11^. Common challenges in individuals with FXS and most other NDDs include anxiety, irritability, social interaction difficulties, sleep problems, speech and language impairments, aggression, motor skill deficits, learning difficulties, etc^12–15^. Notably, many of these features are shared across multiple NDDs, with overlapping molecular mechanisms and functional domains^7^. Thus, FXS provides a valuable model for investigating broader neurodevelopmental pathways and therapeutic targets. Despite advances in preclinical and clinical research, no targeted treatment is currently approved for FXS^16^. Although the influence of the GM on brain function was first demonstrated over a decade ago^17^, the specific role of the GM in FXS remains largely unexplored.

Given the limited availability of effective interventions for FXS and related NDDs, novel therapeutic strategies are urgently needed. Modulating the GM, e.g., through probiotics, fecal microbiota transplantation (FMT), or personalized diets, has emerged as a promising approach. Probiotics, in particular, show potential to alleviate NDD-related symptoms and improve quality of life by reshaping the GM and its communication with the central nervous system^18^.

This pilot study aimed to evaluate whether daily administration of a probiotic mixture containing *Lactobacillus casei*, *Lactobacillus salivarius*, and *Bifidobacterium breve* for 12 weeks would modulate the GM and lead to measurable behavioural improvements in children with FXS. We hypothesised that probiotic-induced alterations in the gut microbiota would be associated with improvements in behavioural domains such as irritability, communication, and socialisation.

## Methods

### Study design

This was an open-label trial without masking (ClinicalTrials.gov ID: NCT06279858, the date of first registration: 22/02/2024), in which each participant orally received a probiotic mixture, once a day, for 12 weeks. Given that an individual’s microbiome is highly personalized, a single-group assignment was chosen as the study design. Participants acted as their own controls by measuring microbiome and other parameters before and after taking the probiotics. The study enrolled participants between January 1 and June 30, 2024, and included three visits: (i) baseline (Visit 1, when all participants began taking the probiotic), (ii) 6-week follow-up (Visit 2), and (iii) 12-week follow-up (Visit 3). The study and all research protocols were conducted in accordance with the Institutional Review Board (IRB) of the Special Hospital for Cerebral Palsy and Developmental Neurology (SHCPDN) in Belgrade, Serbia, which served as the participating site. Written informed consent was obtained from parents/legally authorized representatives prior to study initiation. The detailed study protocol is available at: https://clinicaltrials.gov/study/NCT06279858?locStr=Serbia&country=Serbia&cond=Fragile%20X%20Syndrome&rank=1.

At baseline, a detailed medical history (including presence of ASD and IQ scores), physical and neurological examination and study’s assessments (outcome measures) were carried out, with all medications and medical problems documented for all participants. Specifically, information regarding early developmental milestone delays was obtained retrospectively from existing clinical records and parental interviews. IQ scores were also collected from clinical records and were obtained no more than six months prior to trial initiation. All participants were already registered patients of the SHCPDN, where basic anamnesis, including developmental history, is systematically collected and stored in the local clinical database.

In the first 4 weeks, each patient received a weekly call to evaluate tolerability of the probiotic and any adverse events (AEs).

Visit 2 was organized after 6 weeks and final visit 3 after 12 weeks of study period. Any change in medications was documented during visits. The examination and documentation of AEs, were repeated at each visit, while CGI-I was scoring at visit 2 and final visit 3. Outcome measures were repeated at the final follow-up visit at 3 months/end of treatment. Stool samples were collected twice, at the baseline and final visits. Analyses of microbiome were performed once, at the end of the study, when all samples were collected and sent to the selected laboratory. The complete schedule of all study procedures is also listed in Additional file 1.

### Participants

Eligible participants were children of both sexes, aged 3 to 18 years, diagnosed with FXS and carrying a full mutation of the *FMR1* gene. Basic demographic information was collected on all study subjects at the screening/baseline visit. The presence or absence of ASD was confirmed using the Autism Diagnostic Observation Schedule, Second Edition (ADOS-2), based on clinical records from the SHCPDN, with assessments performed within six months before enrolment. In addition, participants were required to be on stable pharmacological and non-pharmacological treatments for at least 60 days prior to trial initiation, meaning that no changes were made in the type, dosage, or frequency of medications, nor in the intensity or type of behavioral, educational, or therapeutic interventions during this period. Also, their diet was remained stable along the study period (12 weeks). A stable diet referred to the absence of any major changes in participants’ usual eating patterns, food types, or use of nutritional supplements during the 12-week study period. This information was obtained through parental report at each study visit.

Exclusion criteria included the current use of antibiotics or antibiotic use in the last two months (not counting topical antibiotics), any changes in medications, nutritional supplements, or non-pharmacological therapies within 60 days prior to screening. Participants were also excluded if they had a concomitant medical illness or abnormal laboratory test results that, in the investigator’s judgment, would contraindicate study participation (e.g., inflammatory bowel diseases).

### FXS genetic testing

As mentioned above, all included participants were previously diagnosed with FXS. Genomic DNA was extracted from buccal swabs and/or peripheral blood samples using GeneJET Whole Blood Genomic DNA Purification Mini Kit (Thermo Fisher Scientific, USA). Genetic testing for the presence of CGG expansion in the *FMR1* gene was performed using AmplideX^®^ PCR/CE *FMR1* Kit (Asuragen, Austin, TX, USA)^19,20^. In brief, 10–50 ng of genomic DNA was used for amplification reaction. Amplified products were resolved by capillary electrophoresis performed on the ABI3500 Genetic Analyzer (Applied Biosystems by Thermo Fisher Scientific, USA) using POP-4 polymer and a 36 cm capillary. GeneScan™ 600 LIZ™ dye Size Standard v2.0 (Thermo Fisher Scientific, USA) was used as an internal size standard. Capillary electrophoresis data were analyzed using GeneMapper™ ID-X v1.6 (Thermo Fisher Scientific, USA).

### Study drug and justification of probiotic selection

All participants received probiotic mixture (Imunolak^®^, SaveHealth, Serbia) which contains: (i) *Lactobacillus casei* BL 2401 (40%), (ii) *Lactobacillus salivarius* BL 2201 (40%) (iii) *Bifidobacterium breve* BL 3406 (20%). Total amount is 5 × 10^9^ CFU in one HPMC capsule, at the end of the shelf life. These strains are registered and preserved in the French National Collection of Cultures of Microorganisms (CNCM, Collection Nationale de Cultures de Microorganismes). They are on EFSA’s QPS (Qualified and Presumption of Safety) list and are considered safe for use in food and dietary products. Probiotic was administered orally once daily for 12 weeks to children. Adherence to probiotic intake was monitored and verified by parents/caregivers throughout the study.

We selected the probiotic formulation containing *Lactobacillus casei* BL 2401 (40%), *Lactobacillus salivarius* BL 2201 (40%), and *Bifidobacterium breve* BL 3406 (20%) based on mechanistic plausibility and evidence from related fields. These strains belong to genera long recognized for their potential modulatory effects on the gut–brain axis through enhancement of intestinal barrier function, production of neuroactive metabolites, immunomodulation, and regulation of inflammation (e.g. *Lactobacillus*,* Bifidobacterium*)^21,22^. For example, *L. casei* has been shown in animal models to modulate oxidative stress, neurotransmitter levels, and cognitive outcomes via gut-brain signaling pathways^23^. *B. breve* strains have also demonstrated neuroprotective and psychobiotic effects by attenuating neuroinflammation in preclinical models^24^. Although there is no published clinical trial of these specific strains in FXS, their inclusion is justified by prior findings in neurodevelopmental and neuropsychiatric contexts and by their biological relevance to mechanisms implicated in FXS (e.g., gut permeability, inflammation, metabolite signaling). Although neurotransmitters were not directly measured in this pilot study, their mention reflects the established mechanistic relevance of these pathways to the gut–brain axis and provides a biological rationale for probiotic strain selection.

### Safety assessments

Information on adverse effects (AEs), vital signs, height, weight, physical and neurological exams, behavioural/psychiatric assessment, and concomitant medications was collected at every visit. Routine blood tests for haematology/chemistry (complete blood count, comprehensive metabolic panel, and cholesterol, LDL, and triglyceride) were monitored during the study.

### Clinical efficacy assessments

Efficacy assessments were administered to all participants at multiple times throughout the study. All behavioral measures were based on parental reports, either directly or indirectly, through clinician ratings derived from parental input (e.g., the Clinical Global Impression (CGI) scales).

Clinical efficacy assessments included:

(i) primary outcome: the Vineland Adaptive Behaviour Scales-Third Edition (Vineland-3) was administered at baseline and study endpoint to assess adaptive functioning across communication, daily living skills, socialization, and motor skills. Vineland-3 was conducted via parent interview, by a trained researcher during study visits. Higher Vineland-3 scores indicate better adaptive functioning, while lower scores reflect greater difficulties in daily life^25^;
(ii) secondary outcomes:

- Clinical Global Impression-Severity (CGI-S) and Clinical Global Impression-Improvement (CGI-I) as standard assessments for medication studies because it allows the clinician to utilize the history from the parent and incorporate it into a clinical rating for the clinical follow up of the patient through the treatment trial. CGI scales were rated by a clinician, during study visits, based on parental report and direct observation. CGI-S was assessed at baseline, while CGI-I was assessed at study endpoint (12 weeks) to evaluate overall severity and change over time. In the initial evaluation of the patient, the CGI-S (severity) was used to judge the severity of the symptoms with a scale of normal, not at all ill; borderline ill; mildly ill; moderately ill; markedly ill; severely ill; or among the most extremely ill. CGI-I assessed improvement or worsening of symptoms with a scale of very much improved; much improved; minimal improvement; no improvement; minimally worse; or very much worse. Lower CGI-I scores indicate greater improvement, whereas higher scores indicate worsening of the patient’s condition^26^;
- Aberrant Behaviour Checklist-Community (ABC-C) in FXS (ABC-C_FX_), a parents- or caregivers-reported measure, rates behaviours from 0 “not a problem at all” to 3 “the problem is severe in degree” across 54 of the items resolve into 6 subscales (irritability, lethargy, social avoidance, stereotypic behaviour, hyperactivity, and inappropriate speech). ABC-C_FX_ scales were completed by parents at the study’s site during baseline and study endpoint (12 weeks). A decrease in ABC-C_FX_ total and subscale scores from baseline indicates improvement in behavioral symptoms, whereas an increase would suggest worsening^27,28^;
- Pediatric Quality of Life Questionnaire (PedsQL) was administered at baseline and study endpoint, at the study site, to measure quality of life across physical, emotional, social, and school functioning domains. This questionnaire was completed by parents. Higher PedsQL scores indicate better quality of life, while lower scores reflect greater impairment^29^.
- Child Sleep Habits Questionnaire (CSHQ), completed by parents, at baseline and study endpoint at the study site, as a comprehensive tool designed to evaluate the sleep patterns and potential sleep difficulties in children. Parents rated the frequency of specific sleep behaviors over a typical recent week on a 3-point scale (usually: “rarely,” “sometimes,” “usually”). Eight key items were chosen to capture the most relevant domains for children with FXS: sleep onset delay (“child falls asleep within 20 minutes”), bedtime resistance (“child struggles at bedtime”), night wakings (“frequent night-time awakenings”), sleep-disordered breathing (“child snores loudly”), parasomnias (“child grinds teeth” and “child wets the bed”), restlessness during sleep (“child is restless at night”), and daytime sleepiness (“child seems tired in the morning”). Higher scores indicate more frequent sleep problems, and the total score provides an overall measure of sleep disturbance^12,30^.

### Stool samples collection

Stool samples from all participants were collected twice, at the baseline and final visits. The samples were stored in the DNA/RNA shield (Zymo Research, Irvine, CA) at −20 °C.

### DNA extraction from stool samples, library preparation, and metagenomic sequencing

Briefly, stool samples were first homogenized, and 500 µl were transferred to the PowerBead Pro tube from the Qiagen PowerFecal Pro DNA kit. Samples were subjected to bead-beating on a FastPrep-24 machine at 6.5 m/s speed, for 15 s, three times, then DNA was extracted according to the manufacturer’s instructions. DNA-libraries were prepared from extracted DNA with a modified Illumina Nextera XT library protocol^31^. Sequencing was performed with 150 bp paired-end reads on an Illumina NextSeq 2000 (Illumina, San Diego, CA, USA). Raw sequencing data (fastq files) reads were submitted to the European Nucleotide Archive (ENA) under the Bioproject number PRJEB89837 (accession numbers are given in Additional file 6).

### Bioinformatic processing, taxonomic and resistance gene profiling

To remove human read contaminations, resulting reads were mapped against the human reference genome hg19^32^ with BBMap^33^ (minid = 0.6 k = 14 usemodulo bwr = 0.16 fast minhits = 1 qtrim = lr trimq = 20 untrim kfilter = 0 maxsites = 1), and mapped reads were discarded. Afterwards, we used fastp^34^ to remove duplicated and low quality reads (--average_qual 30 --length_required 100 --trim_front1 10 --trim_tail1 4 –dedup). Taxonomic profiling was then performed with metaphlan v4.0^35^ with default settings and vOct22 database. For subsequent analysis, we considered all species with a minimum relative abundance of 0.001% in at least 20% of all samples.

Presence and absence of antimicrobial resistance genes were detected with kma heuristic mapping^36^ against the Comprehensive Antibiotic Resistance Database (CARD) v329_WILDCARD_v402^37^. We only considered genes as present with a minimum coverage of 10x, a minimum pairwise identity of 98% and a minimum target coverage of 98%. Pathway profiling was performed using humann3^38^, with absolute abundances normalized using the relab method.

### Statistical analysis

For clinical assessment analyses, the statistical analyses were performed using SPSS version 26.0 software (SPSS Inc., Chicago, IL, USA). Data are presented as mean ± SD for continuous variables and number (percentage) for categorical variables. The normality of the data was assessed using the Shapiro-Wilk test. Within-subject changes over time were analysed using paired-sample t-tests or Wilcoxon signed-rank tests for non-normally distributed data. Changes in categorical data between time points were analysed using the McNemar-Bowker test. The *p*-values less than 0.05 were considered statistically significant. Cohen’s d was calculated to assess the effect sizes for changes in continuous variables between baseline and follow-up assessments. Cohen’s d was calculated using the following formula: $\:d=\frac{\text{M}1-\text{M}2}{\text{S}\text{D}\:\text{p}\text{o}\text{o}\text{l}\text{e}\text{d}}$, where: M1​ and M2​ are the means of the variable at baseline and follow-up, respectively. SD pooled​ is the pooled standard deviation, calculated as: $\:SDpooled=\:\sqrt{\frac{{SD1}^{2}+{SD2}^{2}}{2}}$ where SD1​and SD2​ are the standard deviations at baseline and follow-up, respectively. Effect sizes were interpreted based on standard thresholds: 0.2: Small effect; 0.5: Medium effect and 0.8 or greater: Large effect.

Statistical analysis of microbial communities was performed with R v4.4.2^39^, data was visualized with ggplot2 v3.5.1. Alpha diversity (i.e. Richness, Shannon, Simpson) and beta diversity (i.e. Bray Curtis, Jaccard) were analysed with vegan v2.6–8. An indicator species analysis was performed with indispecies v1.7.15 and the function “IndVal.g”. Hierarchical cluster analysis was performed with hclust. Heatmaps were calculated with pheatmap v1.0.12. Shapiro Wilk test was applied to test for normality, and Levene’s test for homogeneity of variances. Accordingly, groups were compared with Wilcoxon or paired t-test. Taxonomic and pathway associations with the probiotic intervention were assessed with Maaslin2 v.3.2^40^ using general linear models and including participant ID as random effect. Taxonomic and pathway relative abundance data was log-transformed. We considered only pathway features with a minimum abundance of 0.01% and at least 20% non-zero values across the dataset. Multiple testing correction was applied using the Benjamini–Hochberg false discovery rate (FDR), with results considered significant at p(corr) < 0.05. Matrices were compared with Permanova. Best practice guidelines were followed for species networks constructions^41^. Mean pairwise Jaccard similarity between samples was ~0.39 for both baseline and post-intervention datasets. Species networks were calculated with the sparCC function in SpiecEasi (v1.1.3) based on log-transformed relative abundance tables using the log1p() function to account for compositional and zero-inflated data. SparCC thresholds between 0.1 and 0.3 were tested with 100 iterations to ensure stability and robustness of the correlation estimates, and to refine the networks. We used igraph v2.1.2 to visualize the networks and to compare network properties, including degree centrality (number of connections per node), closeness centrality (distance of a node to all other nodes), betweenness centrality (bridging role of a node between other nodes), and clustering coefficients (tendency of nodes to form clusters)^42,43^.

## Results

### Study cohort

The study included 15 participants (93.3% male) who received daily probiotics comprising *Lactobacillus casei*,* Lactobacillus salivarius*, and *Bifidobacterium breve* for 12 weeks. For participant #14 the follow up study visit could not be performed. Participants’ median age was 9 years (inter quartile range (IQR) 4–13 years and standard deviation (SD) 5.0 years) with a mean body mass index (BMI) of 19.8 (SD 4.8). The developmental milestones were delayed, with independent sitting achieved at a mean age of 8.8 months (SD 1.8 months), crawling at 10.3 months (SD 2.6 months), and walking at 16.7 months (SD 3.4 months). First spoken words were observed at a mean age of 3.4 years (SD 0.5 years), and toilet training was completed at 3.6 years (SD 1.7 years). At the baseline, cognitive assessments revealed a mean total IQ of 43.4 (SD 12.6), while the mean CGI-S score was 3.3 (SD 0.6), with 33.3% of participants demonstrating moderate impairment. According to the ADOS-2 assessment, none of the participants with FXS fulfilled the diagnostic criteria for comorbid ASD.

### Changes in behavioural and functional outcomes

Higher ABC-C_FX_ scores indicate more severe behavioral symptoms, while lower scores reflect improvement. Here, we observed a medium improvement of −3.9, SD ± 5.2 (*p* = 0.027 Cohens’s d = 0.48) over the study period in the ABC-C_FX_ domain of irritability, while other ABC-C_FX_ domains showed trends of reduction, though not statistically significant (*p* > 0.11, Table 1).

In the Vineland adaptive behaviour scales, we observed statistically significant improvements over the study period in communication with a small increase of 1.7 ± 2.5 (Cohens’s d = 0.12, *p* = 0.022) (Table 1). Likewise, socialization improved slightly by 1.4, SD ± 2.1 (Cohens’s d = 0.09, *p* = 0.033), and adaptive behaviour composite scores slightly improved by 1.3 ± 1.4 (Cohens’s d = 0.09, *p* = 0.004).

Daily living skills showed a slight increase, but the change was not statistically significant. The Pediatric Quality of Life Inventory revealed trends of improvement (e.g., PedsQL scores were higher) across domains presented in Table 1. Emotional functioning showed a notable increase, and school functioning also demonstrated a marked increase. However, these changes did not reach statistical significance. Other domains, such as physical functioning and social functioning, demonstrated minor improvements that were not statistically significant.

**Table 1:** Changes in ABC-C_FX_, Vineland, and PedsQL scores from baseline to 12-week follow-up.

	Baseline testing (mean ± SD)	Follow-up (mean ± SD)	Mean difference (mean ± SD)	Cohens’s d	*p* value
**ABC**					
Irritability	12.7 ± 8.1	9.1 ± 7.0	−3.9 ± 5.2	0.48	**0.027**
Hyperactivity	9.7 ± 3.9	8.0 ± 4.8	−1.9 ± 5.1	0.39	0.198
Lethargy	6.3 ± 6.2	6.3 ± 6.2	−0.3 ± 3.8	0.00	0.813
Social avoidance	2.4 ± 3.3	2.2 ± 2.9	−0.4 ± 2.2	0.06	0.589
Stereotypy	5.9 ± 4.2	5.1 ± 3.4	−1.0 ± 3.2	0.21	0.265
Inappropriate speech	4.3 ± 3.4	3.4 ± 2.8	−1.1 ± 2.5	0.29	0.112
Total score	42.5 ± 22.9	35.3 ± 21.0	−8.4 ± 16.3	0.33	0.074
**Vineland-3**					
Communication	40.9 ± 14.9	42.6 ± 14.1	1.7 ± 2.5	0.12	**0.022**
Daily living skills	57.6 ± 14.0	58.4 ± 14.1	0.7 ± 2.3	0.06	0.273
Socialization	54.8 ± 15.3	56.1 ± 14.9	1.4 ± 2.1	0.09	**0.033**
Adaptive behaviour composite	51.9 ± 13.6	53.1 ± 13.4	1.3 ± 1.4	0.09	**0.004**
**PedsQL**					
Physical functioning	78.8 ± 26.7	80.8 ± 22.2	2.0 ± 19.7	0.08	0.709
Emotional functioning	72.5 ± 21.7	81.8 ± 19.7	9.3 ± 15.9	0.45	0.073
Social functioning	50.3 ± 19.8	56.1 ± 21.8	5.7 ± 15.8	0.28	0.199
School functioning	59.6 ± 19.1	70.4 ± 15.3	10.7 ± 19.5	0.62	0.060

ABC - Aberrant Behaviour Checklist, SD – standard deviation, PedsQL - Pediatric Quality of Life.

While most participants (11/14) demonstrated reductions in irritability using ABC-C_FX_, caregivers were reporting higher irritability scores for three participants (Additional file 2). Around half of the patients showed improvements for communication (6/14), socialization (7/14), and adaptive behaviour (8/14) using Vineland-3, while only one participant showing a decline in socialization (Additional file 2). These data indicate a positive effect of the probiotic intervention in some participants for the above mentioned behavioural and functional outcomes.

No statistically significant changes were detected across any of the eight assessed sleep-related domains (see Methods section and research protocol), including sleep onset latency, night-time awakenings, bedtime resistance, snoring, bruxism, nocturnal enuresis, restlessness during sleep, and morning tiredness. Responses in each domain were rated by parents on a three-point scale, where “usually” indicated the behavior occurred five to seven times per week, “sometimes” referred to two to four occurrences per week, and “rarely” indicated the issue appeared one time or less per week. Across the study period, the distribution of these frequency ratings remained stable (*p* > 0.05, all), suggesting that probiotic supplementation did not have a measurable impact on sleep patterns within the study timeframe.

Clinical improvement as assessed by CGI-I scores at follow-up visits demonstrated no significant change between the first and second assessments (*p* = 0.881) (Table 2). At 12 weeks, 40% of participants showed “much improvement,” while 26.7% were “minimally improved.”

**Table 2:** Changes in CGI-I scores between first and second follow-ups.

CGI-I	First follow-up *n* (%)	Second follow-up *n* (%)	*p* value
Greatly improved	1 (6.7)	1 (6.7)	0.881
Much improved	5 (33.3)	6 (40.0)	
Minimally improved	4 (26.7)	4 (26.7)	
No change	4 (26.7)	3 (20.0)	
Missing	1 (6.7)	1 (6.7)	

CGI-I - Clinical Global Impression-Improvement.

### Microbiome composition

Next, we sought to investigate possible changes in the microbial composition of the participants’ gut microbiome prior and after intake of the probiotic mixture. Overall, we sequenced from 14/15 participants one baseline (prior probiotic intake) and one follow-up (daily intake of probiotics over a course of 12 weeks) sample. From participant #14 only the baseline sample was available. Human DNA contaminations were overall very low (< 0.1%), and we generated a minimum of 8.5 M paired reads per sample. After human read removal and quality filtering a median of 9.3 M reads (IQR 8.6 M-10.2 M) were available for metagenomic analysis. In individual participant samples, reads from the yeast *Saccharomyces_cerevisiae*, and the archaea *Methanobrevibacter_smithii*,* Nitrosopumilus* SGB14899, and *Methanosphaera_stadtmanae* were identified. However, with the applied thresholds which included species with 0.001% relative abundance in at least 20% of all samples only bacteria were considered for the subsequent analysis. In total, we detected 206 different bacterial species from 121 genera. Most prevalent species were (i.e. as indicated by median relative abundance) *Faecalibacterium_prausnitzii* (10.1%), *Phocaeicola_vulgatus* (6.7%), *Alistipes_putredinis* (2.4%), *Alistipes_onderdonkii* (1.8%), and *Blautia_wexlerae* (1.1%). In a hierarchical cluster analysis, based on the relative abundance data, paired participant samples were clustered (Additional file 3), and as indicated before the microbial communities comprised mainly species with low relative abundances (< 1%).

### Alpha and beta diversity, co-occurrence and antimicrobial resistence (AMR) patterns

**Fig. 1:**
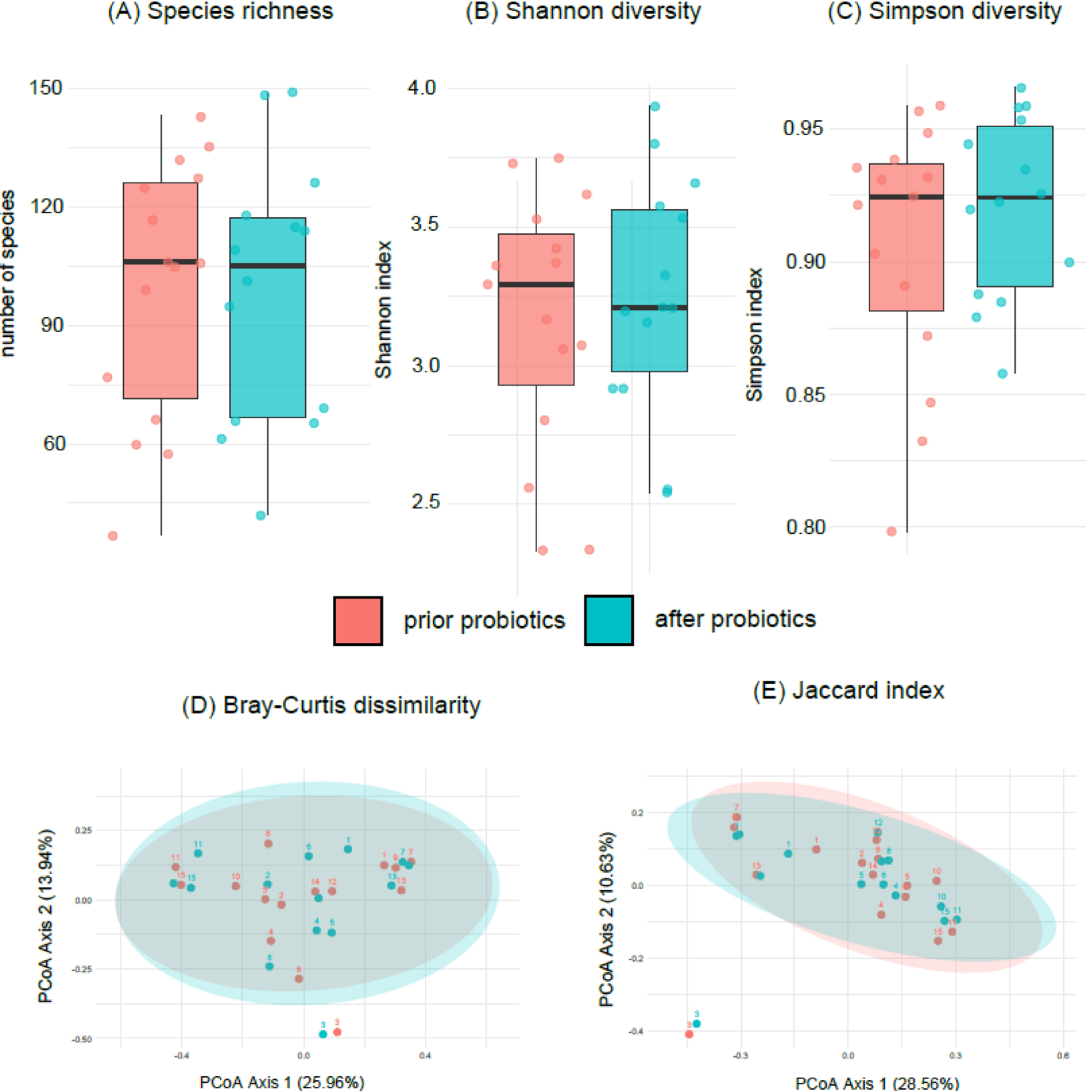
Alpha and beta diversity of the gut microbiome in children with Fragile-X syndrome, prior and after probiotic intake for 12 weeks. No differences in alpha diversity, i.e. species richness **(A)**, Shannon diversity **(B)** and Simpson diversity **(C)** observed. Beta diversity as assessed by Bray-Curtis dissimilarity **(D)** and Jaccard index (E) also did not show differences in species composition.

With regard to changes of the microbial community prior and after the probiotic intervention, we grouped the samples accordingly. Species richness (*p* = 0.93, t-test), Shannon diversity (*p* = 0.58, t-test), and Simpson index (*p* = 0.35, t-test) did not change between the two timepoints (Fig. 1). Beta diversity was assessed by Bray-Curtis dissimilarity and by the Jaccard index (species presence and absence) with no significant differences (*p* = 0.99, and *p* = 1.00, respectively, Permanova) (Fig. 1). An indicator species analysis did not point out individual species which may have been influenced by the probiotic intake. There were also no significant differences in AMR gene presence/absence profiles observed (*p* = 0.97, Permanova), and the number of detected AMR genes did not differ (p = Mann–*p* = 0.91, Whitney U test) between baseline and follow-up samples (Additional file 4). In addition, we constructed co-occurrence matrices, i.e. species (data non shown) and genus presence and absence (Additional file 5) to compare possible interactions patterns prior and after probiotic exposure. Both, on the species and the genus level, significant differences could be observed (*p* < 0.001, Permanova).

### Microbial networks

**Fig. 2:**
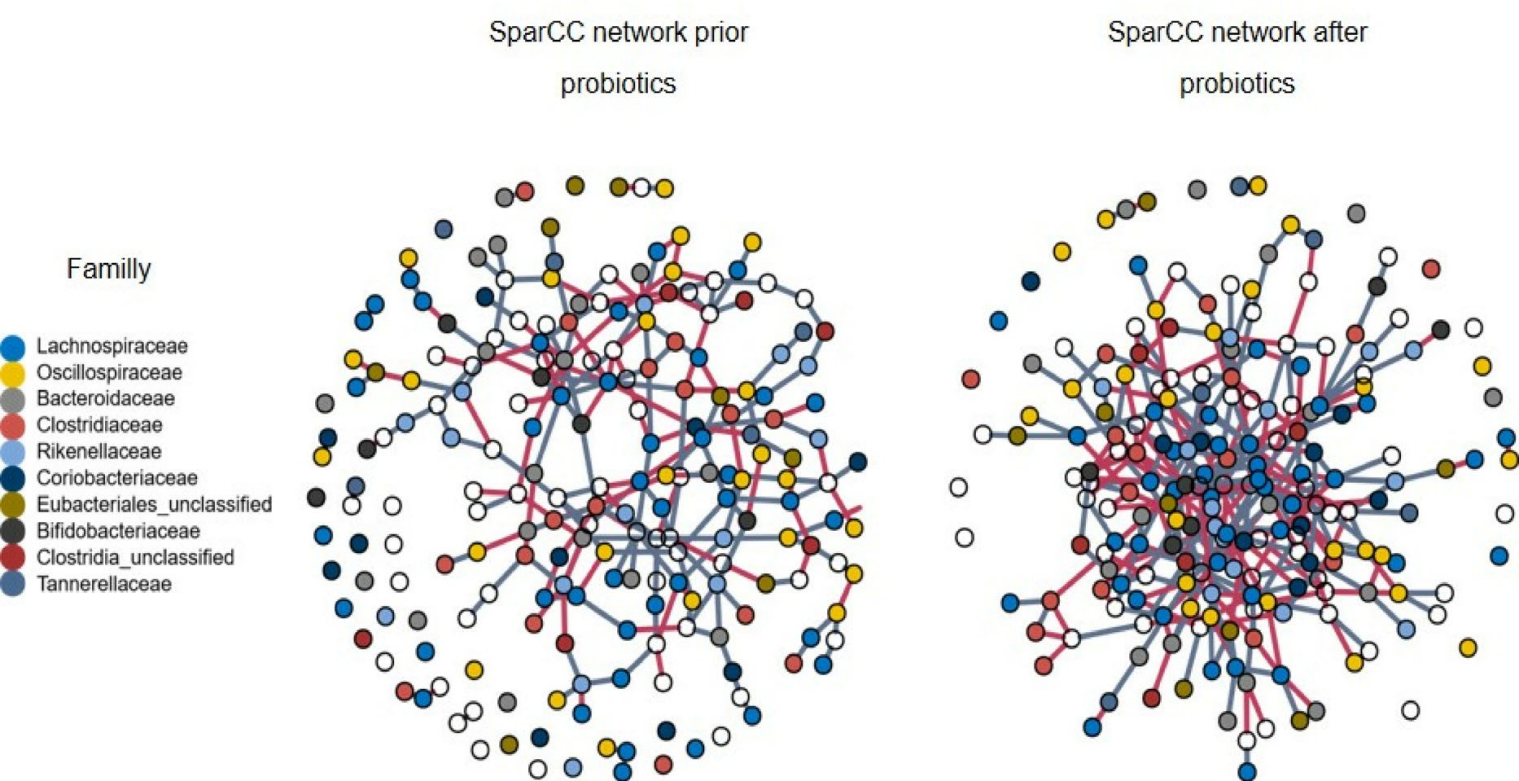
SparCC-inferred microbial species co-occurrence networks before and after probiotic exposure. Nodes represent individual microbial species, with node color indicating the most abundant taxonomic families. Edges represent significant SparCC correlations (≥ 0.2) after 100 iterations, reflecting potential co-occurrence relationships, with edge color indicating the direction of correlation: blue for positive and red for negative correlations. The network following probiotic intervention shows increased connectivity, suggesting enhanced microbial community interactions.

However, co-occurrence patterns based on presence and absence matrices can be influenced by spurious correlations. In order to identify more robust interaction, we performed a network analysis using sparCC on log transformed relative species abundance data. We tested different sparCC threshold between 0.1 and 0.3 which gradually removed weaker interactions (edges) in a species network. All thresholds showed similar trends, associated with higher edge counts (interactions) in the microbial community after the probiotic regimen. In the following we report results for a sparCC threshold of 0.2. The Species network at baseline comprised 213 (SD 17) edges as compared to 289 (SD 15) edges in the follow-up network (*p* < 0.001, Wilcoxon test) (Fig. 2). Likewise, cluster coefficient (*p* < 0.001, Wilcoxon test), betweenness centrality (efficiency to reach other nodes in the network) (*p* < 0.001, Wilcoxon test), degree centrality (number of branches/connections per node) (*p* < 0.001, Wilcoxon test), and closeness centrality (interconnection/bridging of one node on the shortest path between any two nodes) (*p* < 0.001, Wilcoxon test) increased significantly. These results suggest that following probiotic treatment, the microbial network showed an increase in connectivity, indicating a more integrated and interacting microbial community. Although diversity measures remained unchanged, the altered network connectivity highlights significant shifts in microbial interactions.

### Pathway analysis

To assess functional shifts in microbial metabolism, pathway-level associations with the probiotic intervention were analyzed using Maaslin2. Although none of the pathways reached significance after FDR correction (q > 0.05), the top hits showed notable trends in relative abundance between baseline and follow-up samples (Additional file 7). Specifically, the mitochondrial fatty acid biosynthesis initiation pathway (PWY66-429) was enriched after probiotic intake (*p* = 0.018), while the NAD salvage pathway II (PWY-7761) and the L-tyrosine biosynthesis superpathway (PWY-6630) were relatively depleted (*p* = 0.032, and *p* = 0.033). Other pathways enriched in follow-up samples included pyrimidine nucleobase salvage (PWY-7208, *p* = 0.038), starch degradation III (PWY-6731, *p* = 0.039), and isoprene biosynthesis I (PWY-6270, *p* = 0.04), suggesting a potential increase in carbohydrate and energy metabolism.

## Discussion

To our knowledge, this is the first study to evaluate the efficacy of a probiotic mixture containing *Lactobacillus casei*,* Lactobacillus salivarius*, and *Bifidobacterium breve* on behavioural outcomes and GM composition in children with FXS. After 12 weeks of probiotic supplementation, participants showed measurable improvements in irritability, communication, and socialization, which coincided with denser microbial interaction networks.

### Probiotic supplementation and behavioural modulation in FXS

FXS presents a model of genetic and epigenetic dysregulation resulting from CGG repeat expansion and subsequent *FMR1* promoter hypermethylation with a diverse range of neuro-behavioural characteristic^44^. Common spectrum of neuro-behavioural abnormalities includes anxiety as the most prevalent, irritability, impulsivity, hyperarousal, hyperactivity, attentional deficits, aggression, and other manifestations^45,46^. Individuals with FXS are often co-diagnosed with ASD, which affects approximately 50% of males and 20% of females^9,47^. Indeed, evidence suggests that the pathogenetic mechanisms underlying FXS and ASD in the general population partially overlap^48,49^.

Our finding, based on individuals with FXS without ASD, align with previous studies that reported behavioral improvements in children with ASD following probiotic intake, including reductions in irritability and gastrointestinal symptoms^50–52^. It has been also indicated that early-life probiotic supplementation may lower the risk of developing neuropsychiatric disorders later in childhood, potentially through mechanisms beyond just alterations in GM composition^53^. Supporting this idea, maternal supplementation in mice with *Lactobacillus reuteri* during pregnancy has been shown to prevent FXS-like phenotypes in offspring^54^. A meta-analysis of ten studies (522 participants), however, found probiotics only improved overall behavioural symptoms but not core ASD symptoms like social behaviour or communication^55^. Notably, beneficial effects were observed primarily with multi-strain formulations, suggesting that probiotic efficacy may depend on strain diversity and interaction, and study context^55^.

### Impact of Microbiome composition on disease status

The role of GM in FXS pathophysiology remains largely unexplored. In animal models, the maternal microbiota composition has been shown to influence behavioural traits independently of *Fmr1* genotype^56^. In this study, alpha and beta diversity did not differ significantly before and after probiotic supplementation. This finding suggests that the probiotic intervention did not alter the overall community composition or taxonomic diversity but may have influenced how microbial species interact within the community. Such stability in alpha and beta diversity is not unexpected in host-associated microbiomes, particularly over relatively short intervention periods or when probiotics act through functional rather than compositional modulation^57,58^.

This moderate impact of probiotics on overall microbiome diversity aligns with findings from a large-scale metagenomic study in ASD by Yap et al. (2021), which showed that gut microbiome composition was primarily associated with dietary, stool consistency, and age patterns rather than diagnostic status^59^. Similarly, that study emphasized that compositional metrics alone may overlook the complex and interdependent nature of microbial interactions. Similar to the personalized colonization patterns described by Zmora et al. (2018), our network analysis suggests that probiotic supplementation may induce subtle ecological changes that do not shift overall community structure^60^. These effects likely involve shifts in microbial interaction patterns, reflecting changes in metabolic cross-feeding or functional coordination that could influence host behaviour.

### Microbial metabolites and mechanistic links to neuroplasticity

Experimental and clinical evidence supports a functional link between gut microbial metabolism and neurodevelopmental regulation. In *Fmr1* KO2 mice, microbial pathways related to fatty-acid and nucleotide metabolism are altered, and similar metabolic disruptions have been observed in individuals with ASD, suggesting shared microbiome-mediated mechanisms across neurodevelopmental disorders^61^. Human metabolomic studies in ASD further report disturbances in nicotinamide (vitamin B3/NAD salvage), purine, and fatty-acid metabolism, which normalized after microbiota transfer therapy, indicating microbiome-driven modulation of energy and redox pathways^62^. Specific microbial metabolites, such as *p-cresol*, can induce ASD-like behaviours in mice, providing direct evidence that gut-derived metabolites influence neurobehavioral outcomes^63^.

In our study, pathway analysis identified trends toward increased microbial activity in fatty acid biosynthesis, NAD salvage, and starch degradation following probiotic supplementation. Although these changes did not remain significant after multiple-testing correction, their biological relevance aligns with mechanisms linking microbial metabolism to neuronal energy balance and neurotransmitter regulation. Fatty acid and starch degradation pathways contribute to the production of short-chain fatty acids (SCFAs), key mediators of microbiota–gut–brain communication that act through immune, endocrine, and epigenetic routes to influence mood, cognition, and behaviour^64^. Likewise, microbial NAD metabolism supports mitochondrial energy production and redox balance; the NAD salvage pathway replenishes NAD⁺, a cofactor required for sirtuin activity and neurotransmitter biosynthesis^65^.

SCFAs and other microbial metabolites modulate gene expression by promoting histone acetylation and crotonylation, influencing microglial and astrocyte activity and thereby enhancing synaptic plasticity^66–70^. The enrichment of starch degradation and fatty acid biosynthesis pathways in our study may therefore reflect increased SCFA-related metabolic activity consistent with these mechanisms. Beyond SCFAs, other signaling routes, including the tryptophan–kynurenine pathway, GABA/glutamate balance, and neuroinflammatory signaling, are influenced by Lactobacillus- and Bifidobacterium-derived metabolites and have been implicated in FXS and ASD^71–73^.

Finally, the gut microbiome may also influence neurodevelopment through epigenetic regulation, including altered microRNA expression and DNA methylation in brain regions related to cognition and anxiety^74–79^. Although these regulatory processes were not directly assessed here, they provide an additional mechanistic link between microbial metabolism, neuronal plasticity, and behavioural modulation.

### Limitations

The present study indicates a possible link between GM and behaviour in children with FXS, but has several limitations. The small sample size and open-label, single-arm design limit the generalizability of the findings. The absence of a control group and reliance on caregiver-reported outcomes introduce potential reporting and placebo bias, particularly given prior evidence of substantial placebo responses in FXS clinical trials^80^. In addition, multiple comparisons were conducted without Bonferroni correction, and the results should therefore be interpreted as preliminary and hypothesis-generating, requiring confirmation in larger, controlled trials. The 12-week duration may have been insufficient to capture the subsequent microbial shifts, meaning the observed higher connectivity is likely only the beginning of the shift, or behavioural dynamics. This pilot study focused specifically on children with FXS. Therefore, the findings cannot be directly generalized to the broader NDD population.

## Conclusion

This study provides preliminary evidence that probiotic intervention can modulate GM and improve behavioral outcomes in children with FXS. Although exploratory, the observed improvements in irritability, communication, and socialization, along with increased microbial network connectivity and functional shifts in key metabolic pathways, suggest that the microbiome may play a role in the neurobehavioral phenotype of FXS. Further controlled trials with larger cohorts and multi-omics integration are needed to validate these findings and better understand the therapeutic potential of microbiome-targeted interventions in neurodevelopmental disorders.

## Supplementary Information

Below is the link to the electronic supplementary material.

Supplementary Material 1

Supplementary Material 2

Supplementary Material 3

Supplementary Material 4

Supplementary Material 5

Supplementary Material 6

Supplementary Material 7

## Data Availability

The datasets about clinical assessment data generated and/or analysed during the current study are available in the ZENODO repository, [https://zenodo.org/records/15682417] [https://zenodo.org/records/15682417?token=eyJhbGciOiJIUzUxMiJ9.eyJpZCI6ImFjNGMzNjUzLTY4M2QtNGM4OS1iOGMzLWExNDFjYjI2MzI5NiIsImRhdGEiOnt9LCJyYW5kb20iOiJhZGRkZDA2MjY5YzA1ZGM5YzliNmIzYWNiYTBjNmVhNSJ9.6TwI8oM0tynzOYPSRRi7v_70i3d_mnqD_3BfmHhr9QdaKzMqAce4EtPiqmS_MLBB4JPfgR_H8dwq_vSyvPlH_g]. All sequence datasets were submitted to the European nucleotide archive (ENA) and available under bioproject accession PRJEB89837. Runaccessions for all samples are available in Additional file 6.

## Abbreviations

ABC-C: Aberrant Behaviour Checklist-Community
ABC-C_FX_: Aberrant Behaviour Checklist-Community in Fragile X Syndrome
AE: Adverse event
AMR: Antimicrobial resistance
ASD: Autism sprectrum disorder
BMI: Body mass index
CARD: Comprehensive Antibiotic Resistance Database
CGG: Cytosine-Guanine-Guanine trinucleotide repeat
CGI-I: Clinical Global Impression - Improvement
CGI-S: Clinical Global Impression – Severity
CNCM: Collection Nationale de Cultures de Microorganismes
CSHQ: Child Sleep Habits Questionnaire
CNS: Central Nerve System
DNA: Deoxyribonucleic Acid
EFSA: European Food Safety Authority
ENA: European Nucleotide Archive
FDR: False Discovery Rate
FMT: Fecal Microbiota Transplantation
*FMR1*: Fragile X Messenger Ribonucleoprotein 1 gene
FMRP: Fragile X Messenger Ribonucleoprotein
FXS: Fragile X Syndrome
GM: Gut Microbiome
HPA axis: Hypothalamic-Pituitary-Adrenal axis
HPMC: Hydroxypropyl Methylcellulose
IQR: Interquartile Range
IRB: Institutional Review Board
KO: Knockout (mouse model)
MaAsLin2: Multivariate Association with Linear Models v2
miRNA: MicroRNA
NAD: Nicotinamide Adenine Dinucleotide
NDDs: Neurodevelopmental Disorders
PCoA: Principal Coordinates Analysis
PCR/CE: Polymerase Chain Reaction / Capillary Electrophoresis
PedsQL: Pediatric Quality of Life
PERMANOVA: Permutational Multivariate Analysis of Variance
PWY: Pathway
QPS: Qualified and Presumption of Safety
SCFAs: Short-Chain Fatty Acids
SD: Standard Deviation
SHCPDN: Special Hospital for Cerebral Palsy and Developmental Neurology
SPSS: Statistical Package for the Social Sciences
SparCC: Sparse Correlations for Compositional data
