# Supplementary material for: Microbiome modulation and behavioural improvements in children with fragile X syndrome following probiotic intake: A pilot study
Source: Sci Rep. 2025 Dec 5;16:560. doi: 10.1038/s41598-025-29896-1 (PMC12775388; doi:10.1038/s41598-025-29896-1)
Supplement: Supplementary file 3 — Supplementary Material 3 [file 41598_2025_29896_MOESM3_ESM.docx]

The complete schedule of all study procedures

| **Assessment** | **Screening/Baseline**  **Visit 1**  **Week 1, Day 0** | **Visit 2**  **Week 6 +/- 7 days** | **Visit 3**  **Week 12 +/- 7 days** |
| --- | --- | --- | --- |
| Informed Consent | X |  |  |
| Inclusion/Exclusion Criteria | X |  |  |
| Medical History | X |  |  |
| Physical/Neurological Exam | X | X | X |
| Vital Signs | X | X | X |
| Adverse Events^a^ | X | X | X |
| Concomitant Medications^a^ | X | X | X |
| Safety Lab Tests | X |  | X |
| Stool samples collection for microbiome analyses | X |  | X |
| Clinical Global Impression Scale – Severity (CGI-S) | X |  |  |
| Clinical Global Impression Scale–Improvement (CGI-I) |  | X | X |
| Vineland Adaptive Behavior Scales–Third Ed (Vineland 3) | X |  | X |
| Aberrant Behavior Checklist–Community (ABC-C) | X |  | X |
| Pediatric Quality of Life (PedsQL)  Parent Proxy Questionnaire | X |  | X |
| Child Sleep Habits Questionnaire  (CSHQ) | X |  | X |

^a^ Also assessed at phone calls at Weeks 1, 2, 3, 4
