# Supplementary figures and images for: Microbiome modulation and behavioural improvements in children with fragile X syndrome following probiotic intake: A pilot study
Source: Sci Rep. 2025 Dec 5;16:560. doi: 10.1038/s41598-025-29896-1 (PMC12775388; doi:10.1038/s41598-025-29896-1)

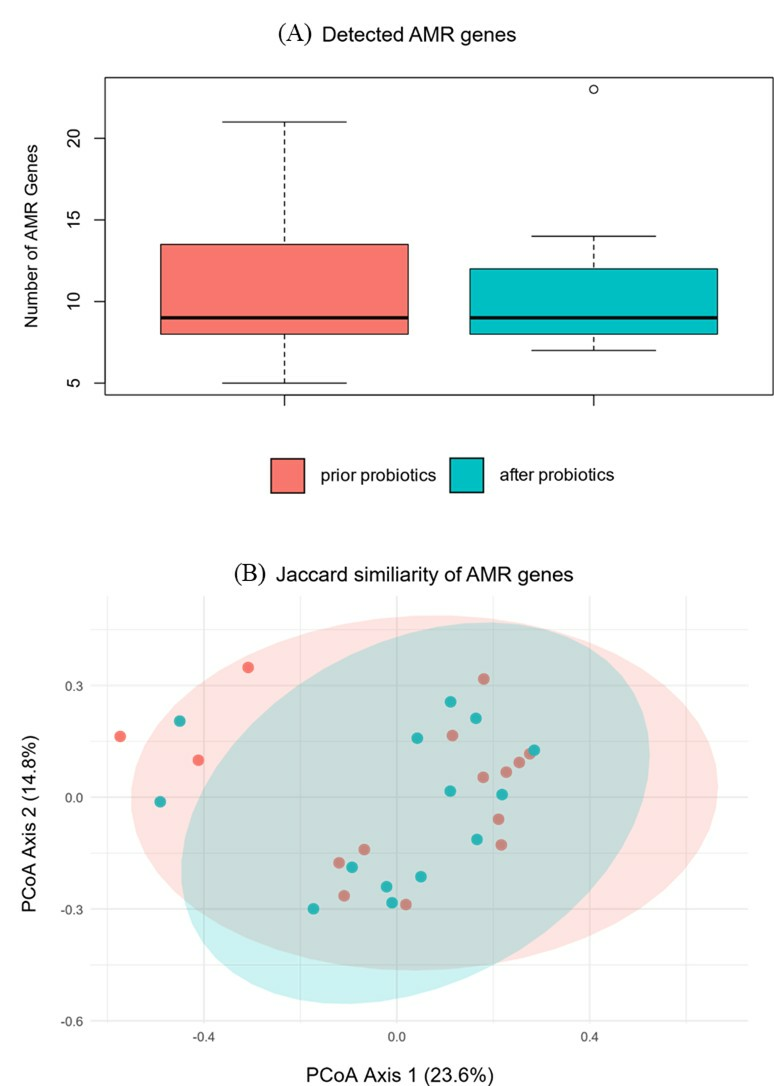

Supplement: Supplementary file 4 — Supplementary Material 4 [file 41598_2025_29896_MOESM4_ESM.tif]

## Supplementary Figure 2

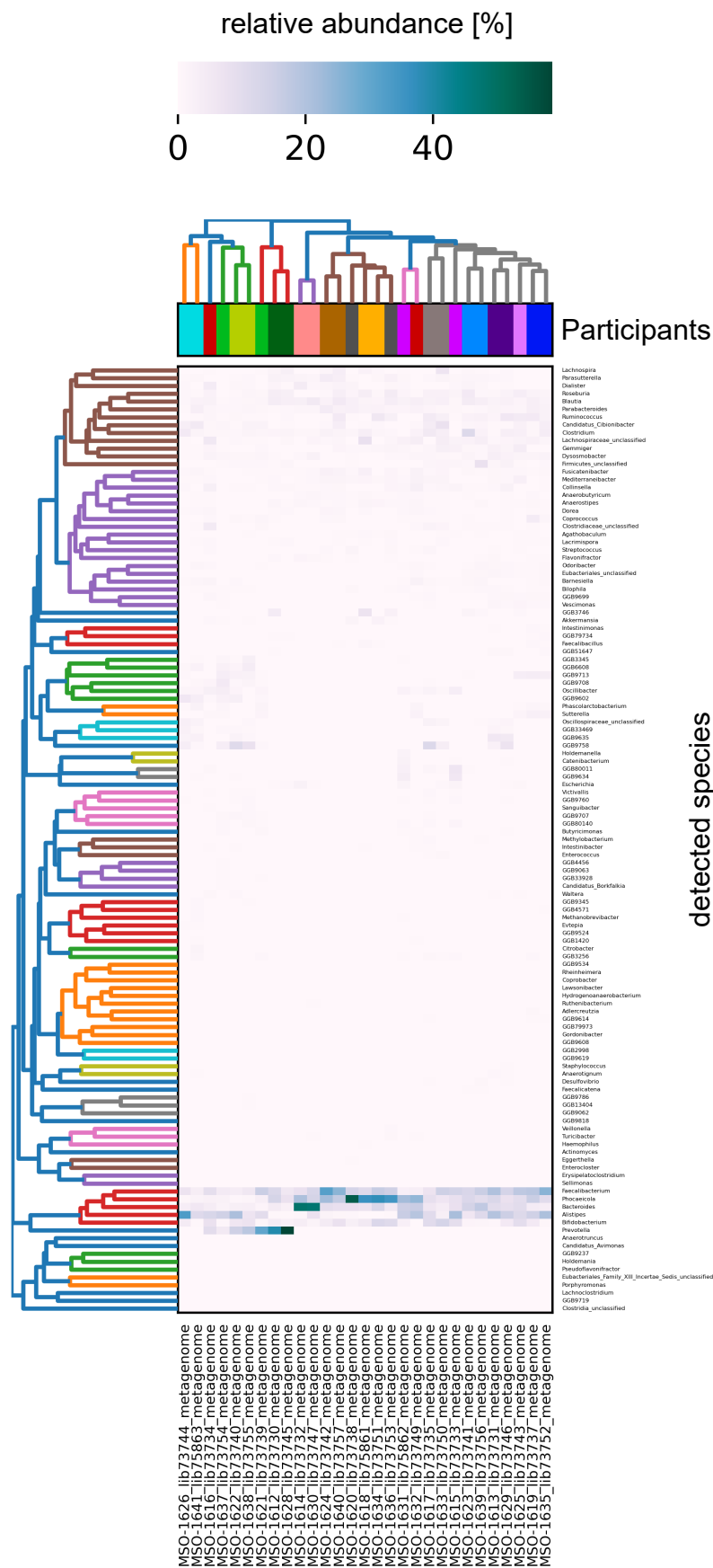

samples

Supplement: Supplementary file 5 — Supplementary Material 5 [file 41598_2025_29896_MOESM5_ESM.pdf]

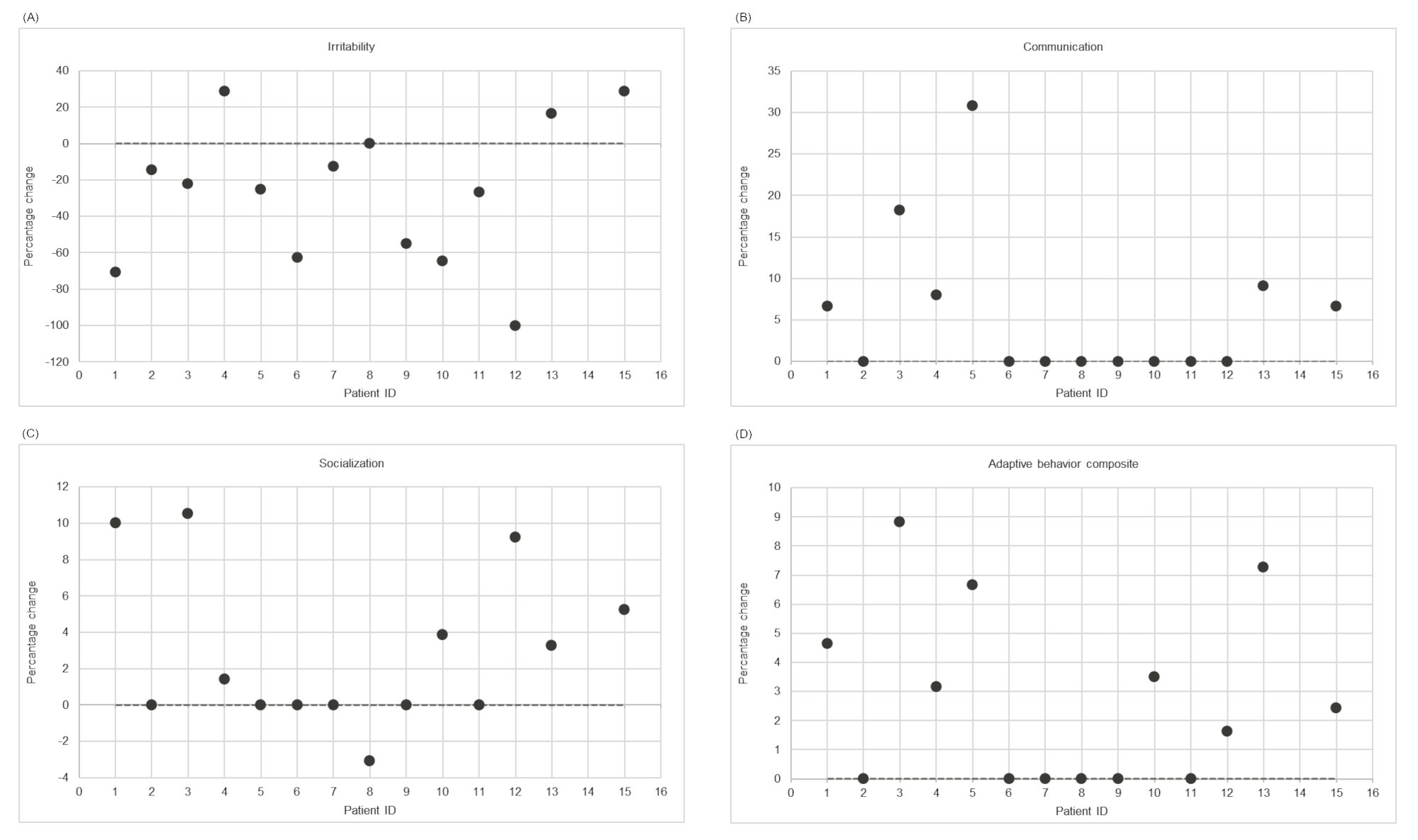

Supplement: Supplementary file 6 — Supplementary Material 6 [file 41598_2025_29896_MOESM6_ESM.tif]

\_\_\_\_\_

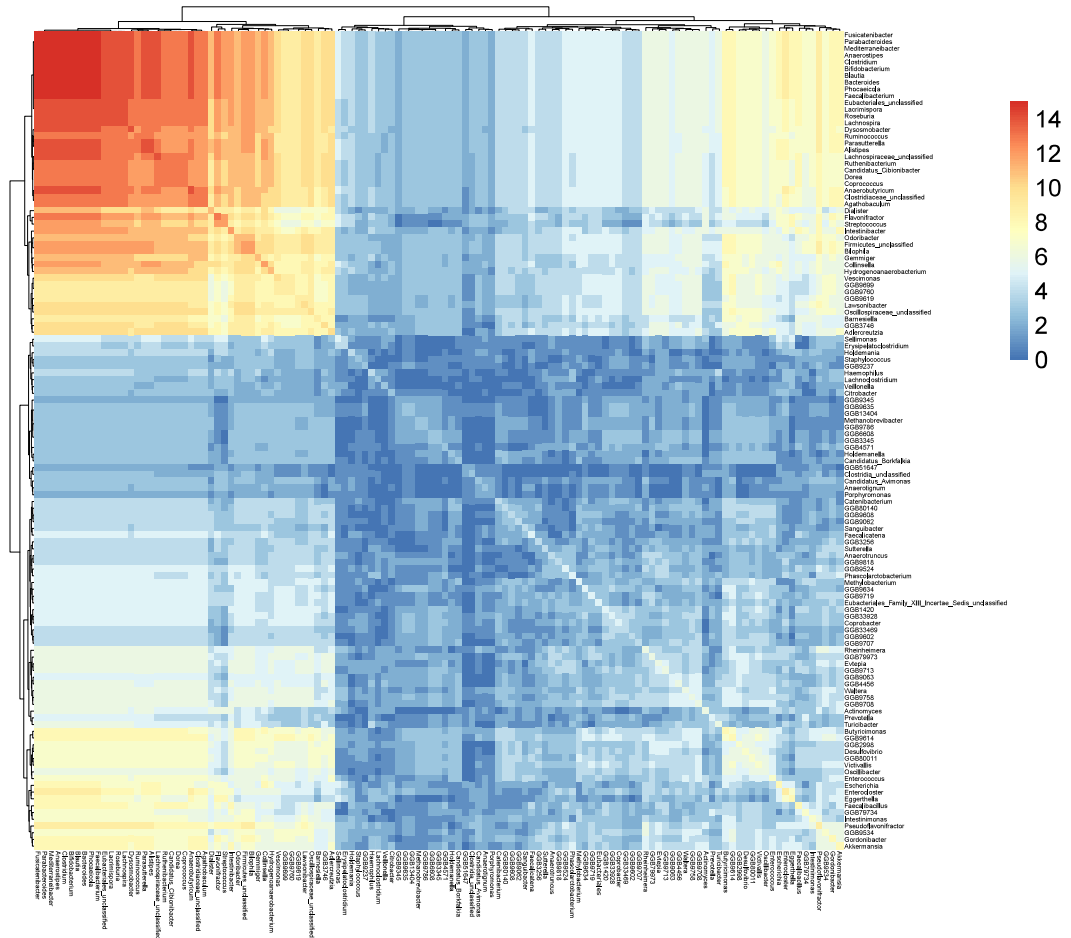

**(B) Pairwise Co-occurrence of Genera after probiotics**

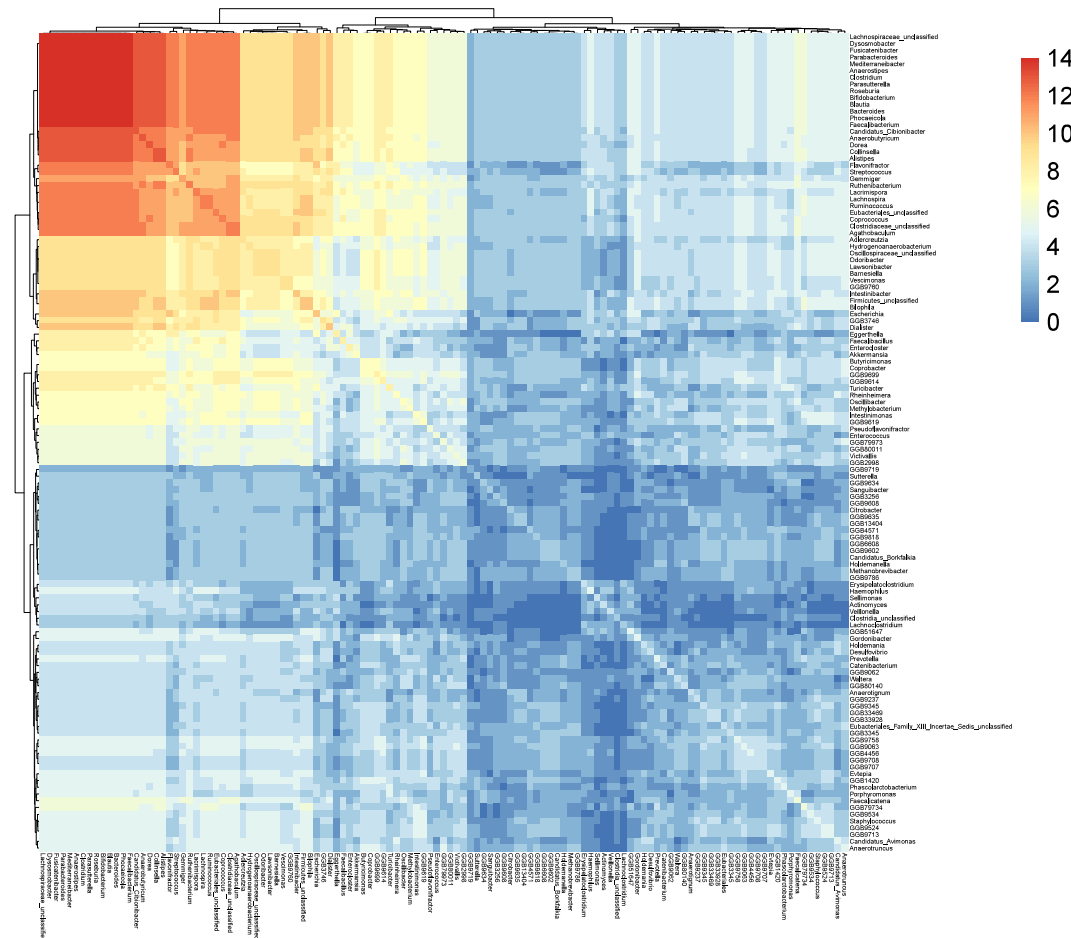

Supplement: Supplementary file 7 — Supplementary Material 7 [file 41598_2025_29896_MOESM7_ESM.pdf]
